# Adenosine-Induced Atrial Fibrillation During Coronary Angiography and Fractional Flow Reserve Procedures

**DOI:** 10.7759/cureus.34328

**Published:** 2023-01-29

**Authors:** Rohit Bhatheja, Shammas Bajwa, Kapil Kapoor

**Affiliations:** 1 Cardiology, AdventHealth Orlando, Orlando, USA; 2 Internal Medicine, AdventHealth Orlando, Orlando, USA

**Keywords:** resting full cycle ratio, coronary angiography, arrhythmia, fractional flow reserve, adenosine

## Abstract

A woman in her sixties presented with a history of progressive shortness of breath, palpitations, and feeling of chest heaviness for the last eight months. To rule out underlying obstructive coronary artery disease, an invasive cardiac catheterization was planned. To assess the hemodynamic significance of the lesion, resting full cycle ratio (RFR) and fractional flow reserve (FFR) values were measured. During this procedure, almost immediately after starting IV adenosine infusion, the patient went into atrial fibrillation which was reversed by IV aminophylline. Awareness of this uncommon effect of adenosine on the cardiac electrical pathways merits knowledge and a thorough follow-up testing of these patients is justified.

## Introduction

Pharmacological testing with agents such as isoproterenol and adenosine is routinely used to characterize the electrophysiological properties of pulmonary vein ectopy [1] and as a diagnostic tool to assess for the presence of non-decremental accessory pathway (AP) conduction before catheter ablation [2]. In interventional cardiology, adenosine is utilized as a vasodilator during FFR (fractional flow reserve) assessment during coronary angiography procedures to assess the hemodynamic significance of coronary stenosis. FFR is the ratio of distal intracoronary pressure to aortic pressure measured during pharmacologically induced hyperemia, while RFR is the non-hyperemic (resting) pressure ratio [3]. Drug-induced atrial fibrillation (AF) is reported to be more likely to be associated with risk factors and comorbidities that commonly coexist with AF, such as advanced age, alcohol consumption, family history of AF, hypertension, thyroid dysfunction, sleep apnea, and heart disease [4]. Episodes of adenosine-induced AF have been reported to be short-lived and transient, because of its short half-life. Although adenosine-induced AF is often clinically inconsequential, it can be of significant clinical importance when an accessory pathway is present. This is one of the rare cases where an intravenous infusion of adenosine (for FFR assessment) induced atrial fibrillation during a coronary angiography procedure.

## Case presentation

A 68-year-old woman presented with a history of Canadian Cardiovascular Society (CCS) class II anginal symptoms for the past eight months. She does not complain of any symptoms of syncope, orthopnea, or claudication. Her past history is relevant for hypothyroidism, migraine, chest radiation for Hodgkin's lymphoma, dyslipidemia, hypertension, coronary atherosclerosis (as evidenced in past by a coronary CT calcium scan), and acid reflux disease. 

During the cardiac work, a pharmacological (regadenoson) nuclear stress test was done. The ECG portion of the pharmacological stress showed no ischemic changes or arrhythmias. Gated perfusion nuclear images showed a moderate area of inferolateral reversible perfusion defect, consistent with ischemia. Echocardiogram revealed normal heart size and functions (ejection fraction around 62%), mild mitral and aortic valvular insufficiencies, and trivial tricuspid regurgitation. 

Due to ongoing symptomology despite medical treatment for presumed angina and coronary artery disease, she was offered invasive cardiac catheterization. At this presentation, planned left heart catheterization and coronary angiography were performed which identified the OM1 branch of the circumflex to have 60-70% proximal stenosis (as this vessel correlated with the ischemic myocardium, we proceeded to the physiological testing with RFR and FFR), diffuse mild disease with no significant stenosis of distal LAD, 30% mild disease of mid circumflex, diffuse 30% disease of mid-right coronary artery (RCA) and mild disease of distal RCA with no high- grade stenosis. A right heart catheterization was also done that revealed normal right-sided hemodynamics.

**Figure 1:**
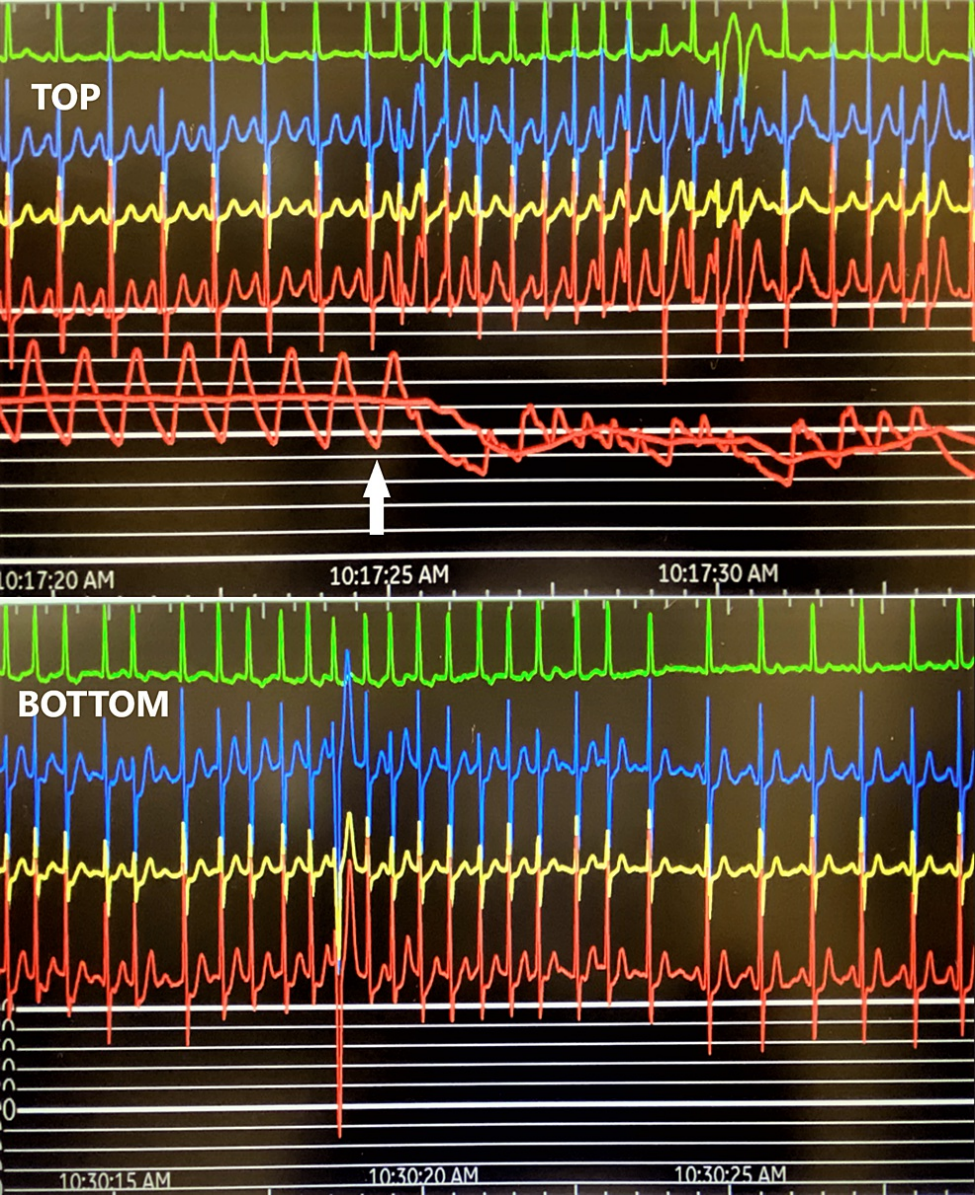
Four-lead electrocardiogram at initial presentation (top) with the onset of atrial fibrillation following administration of adenosine. Return to sinus rhythm in 13 minutes (bottom)

As the patient had significant symptoms of angina and to assess the hemodynamics, further testing using a flow wire was used to measure the resting full cycle ratio (RFR), which came out to be 0.89. Following this, an intravenous infusion of adenosine (140 µg/kg/minute) was administered, and at peak hyperemia (around three minutes) fractional flow reserve (FFR) measured was 0.88, which was consistent with hemodynamically nonsignificant stenosis. Almost immediately after starting the IV adenosine infusion, the patient went into atrial fibrillation with a heart rate of 150 beats per minute. The adenosine infusion was continued uninterrupted for three minutes despite the patient going into atrial fibrillation and we completed the FFR measurement successfully. As the patient continued to have atrial fibrillation with a fast ventricular rate despite stopping adenosine, 75 mg IV aminophylline was given to reverse the effect of adenosine. The arrhythmia lasted for approximately 13 minutes before converting to sinus rhythm (see Figure 1). The patient was observed for a few hours in recovery and had no recurrence during that time. An outpatient mobile cardiac output telemetry (MCOT) monitor showed recurring paroxysmal atrial fibrillation. The patient was started on oral dronedarone 400 mg twice a day and apixaban 5 mg twice a day for rhythm control and stroke prevention, respectively. Her CHA_2_DS_2_-VASc score was 3. In the follow-up visits, the patient did not have any recurrence of atrial fibrillation.

## Discussion

To date, very few cases have been reported involving adenosine-triggered atrial fibrillation during coronary angiography and FFR measurements. To our knowledge, the first case of adenosine-induced arrhythmia was reported by Park et al. [5] during FFR measurements in a case with a middle-aged man requiring cardiac catheterization for exertional chest pain. He had multiple percutaneous interventions in the past. His past history included diabetes, hypertension, and dyslipidemia. Adenosine was infused via the intracoronary route at 70 µg/kg/minute that induced atrial fibrillation and the patient remained in atrial fibrillation for a day that was aborted by IV amiodarone [5]. In another case report, a patient with suspected supraventricular tachycardia in an otherwise normal heart was administered a 6 mg intravenous bolus dose of adenosine after an unsuccessful vagal maneuver attempt, which led to ventricular fibrillation and circulatory collapse that was successfully defibrillated to sinus rhythm. No further arrhythmia was identified on continuous cardiac monitoring [6]. As a routine protocol, our patient (100.4 kg body weight) received a relatively higher adenosine IV dose at 140 µg/kg/minute which could amount to around 42 mg dose in three minutes.

In another study, a spectrum of responses to pulmonary vein ectopy to adenosine after pulmonary vein (PV) isolation was identified in patients undergoing radiofrequency catheter ablation for paroxysmal or persistent symptomatic drug-refractory atrial fibrillation [1]. AF induction following routine adenosine myocardial perfusion stress testing in 0.41% of the patient population in a given year has previously been demonstrated [7]. Additionally, approximately 3% of patients receiving adenosine for termination of supraventricular tachycardia, and up to 12% of patients receiving adenosine during electrophysiologic studies of supraventricular tachycardia develop atrial fibrillation [8]. Another case of a patient with a history of supraventricular tachycardia and myocardial infarction who developed atrial fibrillation (AF) and polymorphic ventricular tachycardia sequentially following the administration of 12 mg of adenosine is described [9].

We report adenosine-induced atrial fibrillation during FFR measurements. Interestingly our patient had no structural heart disease but has a past history of migraine and takes rizatriptan, a serotonin (5-HT) receptor agonist. 5-HT has been shown to cause prolongation of the nodal refractoriness more than atrial-His conduction time which leads to an increase in the excitability index without significant reduction of the ventricular rates during AF [10]. The presence of comorbidities like coronary atherosclerosis, obesity, dyslipidemia, hypertension, and hypothyroidism in our patient may have led to a predisposition for increased sympathetic tone, autonomic dysregulation, and increased sensitivity of the cardiac tissue to adenosine leading to paroxysmal atrial fibrillation that was unmasked by IV adenosine administration during cardiac catheterization [5]. Several reports have indicated that adenosine can generate autonomic activation of PV triggers. Biaggioni et al. [11], suggested that intravenous adenosine administration primarily activates afferent nerves via arterial (or carotid body) chemoreceptors or baroreceptors resulting in sympathetic activation. This is frequently followed by an abrupt shift toward vagal predominance, which may facilitate the induction of AF from the arrhythmogenic site. In addition, adenosine has been shown to favor AF by dispersion and shortening of atrial refractoriness. These effects can be largely explained by the ability of adenosine to increase steady-state outward potassium current.

Currently, in routine clinical practice intravenous adenosine is used to measure FFR in patients with atrial fibrillation and CAD with no reported changes in clinical outcomes (as compared to those patients in sinus rhythm) per our knowledge and detailed research has provided enough confidence to conclude that FFR is minimally affected by changes in hemodynamics [12].

## Conclusions

To conclude, this is one of the rare cases where during a coronary angiography procedure, the use of intravenous adenosine (for FFR assessment) induced atrial fibrillation that was later confirmed with patient monitoring. Detailed history, possible underlying accessory pathways, comorbidities, knowledge, and understanding of drugs that can interact and possibly increase the sensitivity of adenosine on cardiac tissue is very helpful in managing the uncommon adverse effects of this drug. In the right patient population, further outpatient monitoring must be pursued to uncover asymptomatic AF. Rapid reversal and other resuscitative measures must be readily available to counteract uncontrollable arrhythmias.
